# Model-based calculating tool for pollen-mediated gene flow frequencies in plants

**DOI:** 10.1093/aobpla/plw086

**Published:** 2016-12-30

**Authors:** Lei Wang, Bao-Rong Lu

**Affiliations:** Ministry of Education Key Laboratory for Biodiversity and Ecological Engineering, Department of Ecology and Evolutionary Biology, Fudan University, Songhu Road 2005, Shanghai 200438, China

**Keywords:** Biosafety assessment, coexistence, isolation distance, modelling, pollen-mediated gene flow, seed production

## Abstract

The potential social-economic and environmental impacts caused by transgene flow from genetically engineered (GE) crops have stimulated worldwide biosafety concerns. To determine transgene flow frequencies resulted from pollination is the first critical step for assessing such impacts, in addition to the determination of transgene expression and fitness in crop-wild hybrid descendants. Two methods are commonly used to estimate pollen-mediated gene flow (PMGF) frequencies: field experimenting and mathematical modelling. Field experiments can provide relatively accurate results but are time/resource consuming. Modelling offers an effective complement for PMGF experimental assessment. However, many published models describe PMGF by mathematical equations and are practically not easy to use. To increase the application of PMGF modelling for the estimation of transgene flow, we established a tool to calculate PMGF frequencies based on a quasi-mechanistic PMGF model for wind-pollination species. This tool includes a calculating program displayed by an easy-operating interface. Pollen-mediated gene flow frequencies of different plant species can be quickly calculated under different environmental conditions by including a number of biological and wind speed parameters that can be measured in the fields/laboratories or obtained from published data. The tool is freely available in the public domain (http://ecology.fudan.edu.cn/userfiles/cn/files/Tool_Manual.zip (14 December 2016)). Case studies including rice, wheat and maize demonstrated similar results between the calculated frequencies based on this tool and those from published PMGF data. This PMGF calculating tool will provide useful information for assessing and monitoring social-economic and environmental impacts caused by transgene flow from GE crops. This tool can also be applied to determine the isolation distances between GE and non-GE crops in a coexistence agro-ecosystem, and to ensure the purity of certified seeds by setting proper isolation distances among field production plots.

## Teaser

This study provide a model-based calculating tool/software that can accurately calculate pollen-mediated gene flow (PMGF) frequencies of wind-pollinated plant species by including five biological and climatic parameters. The calculating tool can easily be used by any users who are not familiar with mathematical modeling. The required parameters can be measured directly in the field without conducting PMGF experiments, which makes this tool practical to estimate PMGF frequencies. This tool will be helpful to estimate transgene flow and its potential impacts, in addition to determining isolation distances between GE and non-GE crops.

## Introduction

The extensive cultivation of genetically engineered (GE) crops has stimulated worldwide concerns over biosafety issues (Timmons *et al.* 1996; Mehrotra and Goyal 2013). The undesired social-economic and environmental impacts caused by transgene flow from a GE crop to its non-GE varieties (crop-to-crop) and wild relatives (crop-to-wild/weed) are among the most concerned and debated issues (Ellstrand *et al.* 1999; Stewart *et al.* 2003; Andow and Zwahlen 2006; Lu 2016). Crop-to-crop transgene flow may cause adventitious presence (contamination) of transgenes in the non-GE crop varieties. Such transgenic ‘contamination’ of non-GE crop varieties may create regional or/and international trade problems or even legal disputes (Metz and Fütterer 2002). Crop-to-wild/weed transgene flow may result in unwanted environmental impacts (Ellstrand 2003; Lu and Snow 2005; Lu and Yang 2009; Ellstrand *et al.* 2013). It is essential to assess such impacts before commercial cultivation of GE crops. The assessment of transgene flow and its potential impacts includes the estimation of (trans)gene flow frequencies, transgene expression in crop-wild/weed hybrid descendants, and fitness effects caused by transgenes (Stewart *et al.* 2003; Snow *et al.* 2008; Lu and Yang 2009; Lu *et al.* 2016). If the frequency of transgene flow is moderate to high, the expected environmental consequences and impacts might be substantial (Yong and Kim 2001; Rong *et al.* 2005), and *vice versa*. Therefore, the determination of (trans)gene flow frequencies is the first key step to assess the potential social-economic and environmental impacts caused by transgene introgression into wild/weedy relative species (Ellstrand 1992, 2003; Yong and Kim 2001; Lu and Snow 2005; Loureiro *et al.* 2009; Lu and Yang 2009; Ellstrand *et al.* 2013).

Two methods are commonly used to measure the frequencies of pollen-mediated gene flow (PMGF): field experimenting and mathematical modelling. To date, field experimenting is the major method to determine crop-to-crop and crop-to-wild/weed PMGF frequencies at various spatial distance intervals (Song *et al.* 2003*a*; Chen *et al.* 2004; Rong *et al.* 2004, 2005, 2007; Beckie *et al.* 2008). However, generating PMGF data from field experiments are usually time and resource consuming, although reliable PMGF frequencies can be obtained under specific environmental conditions. In addition, PMGF frequencies estimated from a limited number of field experiments may not represent PMGF frequencies of a particular plant species under diverse environmental conditions. As a comparison, mathematical modelling can provide relatively quick estimation of PMGF frequencies for different plant species under diverse environmental conditions (Yao *et al.* 2008; Rong *et al.* 2010; Hu *et al.* 2014). Undoubtedly, mathematical modelling provides a powerful complement of the field-experiment-based method to estimate PMGF frequencies, provided that the model can accurately simulate PMGF (Rong *et al.* 2010; Wang *et al.* 2016).

Many PMGF models have been established (Loos *et al.* 2003; Klein *et al.* 2006; Yao *et al.* 2008; Wang and Yang 2010; Hu *et al.* 2014). However, some of these models essentially simulate the variation patterns/dynamics of PMGF or describe parameters that affect PMGF frequencies (Loos *et al.* 2003; Klein *et al.* 2006). In addition, other PMGF models are also used to assess the maximum PMGF frequencies at a particular spatial distance interval (Gustafson *et al.* 2005; Gaines *et al.* 2007). Such features make these models difficult to be applied directly to predict PMGF frequencies under specific situations. Furthermore, many PMGF models only include sophisticated equations or functions (Yao *et al.* 2008; Wang and Yang 2010; Hu *et al.* 2014), which puzzles the users who are not familiar with the theories and skills of modelling. It is difficult for the users to directly use these models to estimate PMGF frequencies. For example, some PMGF models only included mathematical equations that were generated from two-dimensional Gaussian distribution and double integral to describe PMGF patterns (Yao *et al.* 2008; Hu *et al.* 2014), challenging for common users. The above limitation hinders the application of mathematical modelling for the prediction of PMGF frequencies. Thus, to establish a practical and easy-operating tool that can be used to accurately calculate PMGF frequencies based on an appropriate model will be useful for biologists, managers and regulators. Such a tool will facilitate the assessment and monitoring of transgene flow and its social-economic and environmental impacts (Lu and Snow 2005; Lu and Yang 2009; Lu 2016).

A number of factors should be considered when transforming a theoretical PMGF model into a practical and easy-operating tool to calculate PMGF frequencies. First, an appropriate PMGF model that can accurately estimate PMGF frequencies should be selected for the tool construction. Second, the key parameters used for calculating PMGF frequencies should be easily measured from the target plant species and environment at the site (*in situ*) where the estimation of PMGF frequencies are required, without conducting additional PMGF experiments. Third, a simple and easy human–computer communication/interaction interface that is accessible through the Internet worldwide should be established and ready to use. Therefore, anyone from any part of the world can use this tool to calculate PMGF frequencies under diverse environmental conditions, provided that the required parameters are measured based on field- or laboratory-experiments, in addition to published data.

The objective of this study is to establish a practical tool or programme for the calculation of gene flow frequencies based on an existing PMGF model. To meet such an objective, we need to (i) select an appropriate PMGF model for constructing the tool; (ii) determine suitable and easy-measurable parameters for the PMGF tool calculation and (iii) construct a simple and easy-operating interface for the human–computer dialogs. In addition, the predicting power of this PMGF calculating tool should be tested by comparing the PMGF frequencies obtained from the tool and field experiments. The establishment of this PMGF calculating tool can substantially facilitate biosafety assessment of transgene flow by efficiently and accurately calculating PMGF frequencies under different environmental conditions, in addition to designing spatial isolation distances between GE and non-GE crops in the coexistence farming system, and for certified seed production.

## Methods

### Determination of model and parameters

To calculate gene flow frequencies accurately, without conducting field experiments, we should select a suitable PMGF model with the following features for the tool construction: (i) the model should include biological and climatic parameters that can essentially determine the PMGF frequencies; (ii) generating data or parameters from PMGF experiments is not required during model simulation; (iii) the obtained PMGF frequencies from model simulation should be highly consistent with the field-experimental generated PMGF frequencies.

Similarly, the parameters used for the calculation of PMGF frequencies should meet the following criteria: (i) easily measureable from the environment *in situ* or obtained from published data; (ii) sensitive to determine the change of PMGF frequencies. In addition, the knowledge of interrelationships between PMGF and the parameters should be clearly understood or determined in the previously published studies.

### Construction of the calculating tool

To create a human–computer dialog system with an easy-operating and visualized interface, we decided to construct the calculating tool (programme) for PMGF frequencies using the Visual Basic (VB) language (Microsoft Corporation). Numerical integrations for PMGF calculation followed the Simpson integration method (Davis and Rabinowitz 1979). The outputs of calculated PMGF frequencies were described either as a value (frequency) at a given spatial distance interval, or as a curve (frequencies) at a series of spatial distance intervals in plotting field after all the required parameters were included.

### Case studies: calculating PMGF frequencies

Three wind-pollination crop species were selected for case studies: rice, wheat and maize. This is because published data from the PMGF experiments are available for the three crop species. These data were used to examine the predicting power of the PMGF calculating tool by comparing the results generated by the model simulation and those obtained from the PMGF experiments. In addition, all the biological parameters required by the calculating tool are accessible for the three crop species.

For the required parameters, *pollen diameter* (the diameter of pollen grains) of the three species was determined based on data from published studies (Goss 1968; Chaturvedi *et al.* 1998). The parameter of relative *pollen release height* was determined following the method described in Wang *et al.* (2016). The parameter of *outcrossing rate* was estimated based on the natural hybridization rate (the proportion of hybrid seeds in total seed set) measured at the nearest distances for rice (Messeguer *et al.* 2001; Rong *et al.* 2005), wheat (Griffin 1987; Martin 1990) and maize (Goggi *et al.* 2007; Porta *et al.* 2008). The parameter of *crossability* was determined as an average of 90 % for crop-to-crop gene flow. The parameter of *wind speed* was determined following the actual wind speed measured in the PMGF experiments of case studies.

## Results

### Model and parameters

Following the above criteria, we selected the quasi-mechanistic model described by Wang *et al.* (2016) to construct the PMGF calculating tool. This model included an inverse Gaussian function to describe the pollen dispersal pattern of wind-pollinated plants. The model is shown as follows:

(1) $$F_{\text{A}\text{B}}(x)=t_{\text{B}}\frac{\delta_{\text{A}\text{B}}D_{\text{A}}}{\delta_{\text{A}\text{B}}D_{\text{A}}+D_{\text{B}}}=t_{\text{B}}\frac{\delta_{\text{A}\text{B}}(\phi (x+b)-\phi (x))}{\delta_{\text{A}\text{B}}(\phi (x+b)-\phi (x))+\phi (x-R)}$$

where the *ϕ* is the cumulative distribution function of the inverse Gaussian function (Katul *et al.* 2005) and can be calculated based on three parameters: *pollen diameter*, *pollen release height* and *wind speed* [see equations (2–4) in Wang *et al.* 2016]; *t*_B_ indicates the *outcrossing rate* of a recipient; *δ*_AB_ indicates the *crossability* between a pollen donor and recipient; *x* indicates the variable of *distance to recipient*; *b* indicates the depth of a donor field (determined as infinite for the worst scenario assessment in the calculating tool). Through the replacement of the exponential function in the model of Rong *et al.* (2010) by the function *ϕ*, the model of Wang *et al.* (2016) can easily use the measurable parameters to estimate PMGF frequencies. In addition, this model contained both biological and climatic parameters that can be measured at the field sites where PMGF frequencies are attempted for PMGF calculation, which can provide more accurate results. Furthermore, this model can provide highly consistent PMGF frequencies with those generated from PMGF experiments. Therefore, this model is suitable for constructing the PMGF calculating tool. Detailed information of this PMGF model is described in the published article (open access) by Wang *et al.* (2016) that is available at the link: (http://journals.plos.org/plosone/article/asset?id=10.1371%2Fjournal.pone.014 9563.PDF (14 December 2016)). Apparently, the calculating tool can be used to generate PMGF frequencies through model simulation, independent of a particular PMGF experiment.

For the criteria of easy measurement, five parameters were identified for constructing the PMGF calculating tool. These included the diameter of donor’s pollen, pollen release height, outcrossing rate of pollen recipients, crossability between pollen donors and recipients, and wind speed. Detail description of the five parameters and methods of their measurement are indicated as follows.

*Pollen diameter* (μm). The average diameter of pollen grains from a pollen donor, which can be obtained by directly measuring diameters of 20–30 pollen grains under a microscope.

*Pollen release height* (m). The average vertical differences between the height of updraft pollen (male flowers) from donor plants and flowers of recipient plants (for detail see Fig. 1 in Wang *et al.* 2016). The average height of the updraft pollen can be measured using the pollen trap method (Song *et al.* 2004). Pollen release height can be obtained by measuring the average height of updraft pollen grains and flowers from about 10 plants.

**Figure 1:**
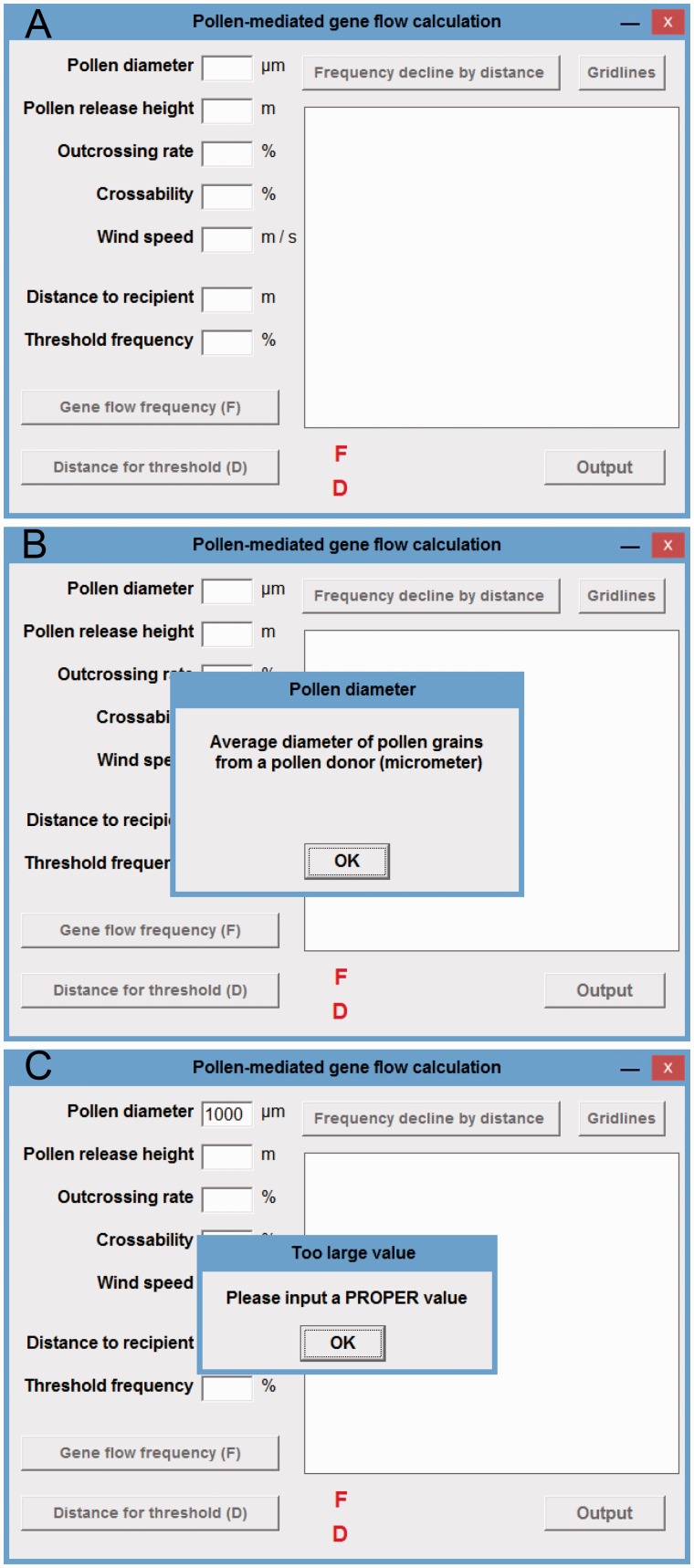
Interface of the calculating tool for PMGF. (A) The initial interface when tool is run; (B) the pop-up window defining the parameter of ‘Pollen diameter’ when clicking the ‘Pollen diameter’ input text; (C) the pop-up window reminding users when improper values are included.

*Outcrossing rate* (%). The probability of mating in plants in which a male gamete in one individual (any plant) fertilizes a female gamete in another individual (recipient) (Waines and Hegde 2003), which can be estimated by hybridization experiments or using molecular markers (Ritland *et al.* 1981; Xia *et al.* 2011).

*Crossability* (%). The ability of two individuals (a donor and a recipient) to cross or hybridize (Schlegel 2003), which can be estimated by measuring the ratio of hybrid seeds generated from artificial crosses between donor and recipient plant species/taxa.

*Wind speed* (m/s). The average horizontal wind speed at the canopy height of donors and recipients, which can be measured using an anemometer at the sites where PMGF frequencies will be estimated.

In addition, the PMGF calculating tool also included two variables: (i) *Distance to recipient* (m) that is defined as the distances from the edge of a donor field to target recipient plants; (ii) *Threshold frequency* (%) that is designed to calculate the spatial isolation distance (m) to guarantee the GE crop contamination under the permitted low-level presence (LLP). The distance to recipients can be determined as the initial distance where the PMGF frequency needs for calculation from the edge of a donor field to recipient plants. The threshold frequency can be determined according to the users’ requirement in different countries or regions. With the five parameters and two variables, the tool can be used to calculate PMGF frequencies for different pairs (donors and recipients) of plant species and a spatial isolation distance (m) required by meeting a particular threshold frequency (%) under various environmental conditions.

### Interface of the calculating tool

The established PMGF calculating tool and a user’s manual can be downloaded from the web site by clicking the link at: http://ecology.fudan.edu.cn/userfiles/cn/files/Tool_Manual.zip (14 December 2016). After clicking, a pop-up window with a download file ‘Tool_Manual.zip’ or download prompt requesting the path to save the zip file will appear. Download the ‘Tool_Manual.zip’ file to a computer and unzip it. An executable file named ‘Calculating tool for pollen-mediated gene flow.exe’ (152 kb) and a PDF file named ‘User's Manual_Calculating Tool for Gene Flow Frequencies’ will be available in the folder. The manual will provide a step-wise guide for users to calculate PMGF frequencies. The executable file for the tool can be directly run by double clicking. After that, an initial interface with all required input textboxes (parameter/variable), output texts including PMGF frequency (*F*) and distance for threshold (*D*), a plotting field and commanding buttons (Fig. 1A) will be displayed. Information or explanation about the parameters and variables included in the tool will be displayed in a pop-up window when clicking the relevant input texts (e.g. pollen diameter) at the left side of the interface (Fig. 1B).

Once all the required data for the five parameters and the ‘distance to recipient’ variable are included in the relevant textboxes, the expected PMGF frequencies of the target plant species can be calculated and displayed by clicking the ‘Gene flow frequency (*F*)’ and ‘Frequency decline by distance’ buttons. Pollen-mediated gene flow frequency (lower side of the interface) at a given spatial distance interval can be calculated by clicking the ‘Gene flow frequency (*F*)’ button. A series of PMGF frequencies (right side of the interface) at a certain distance range can be plotted as a curve by clicking the ‘Frequency decline by distance’ button. Gridlines can be added in the plotting field to estimate PMGF frequencies at particular distance intervals by clicking the ‘Gridlines’ button. In addition, if non-numerical or improper values (e.g. pollen diameter as 1000 μm) are included in the textboxes, this tool will display a pop-up window to warn the users ‘Please input a PROPER value’ (Fig. 1C).

**Figure 2:**
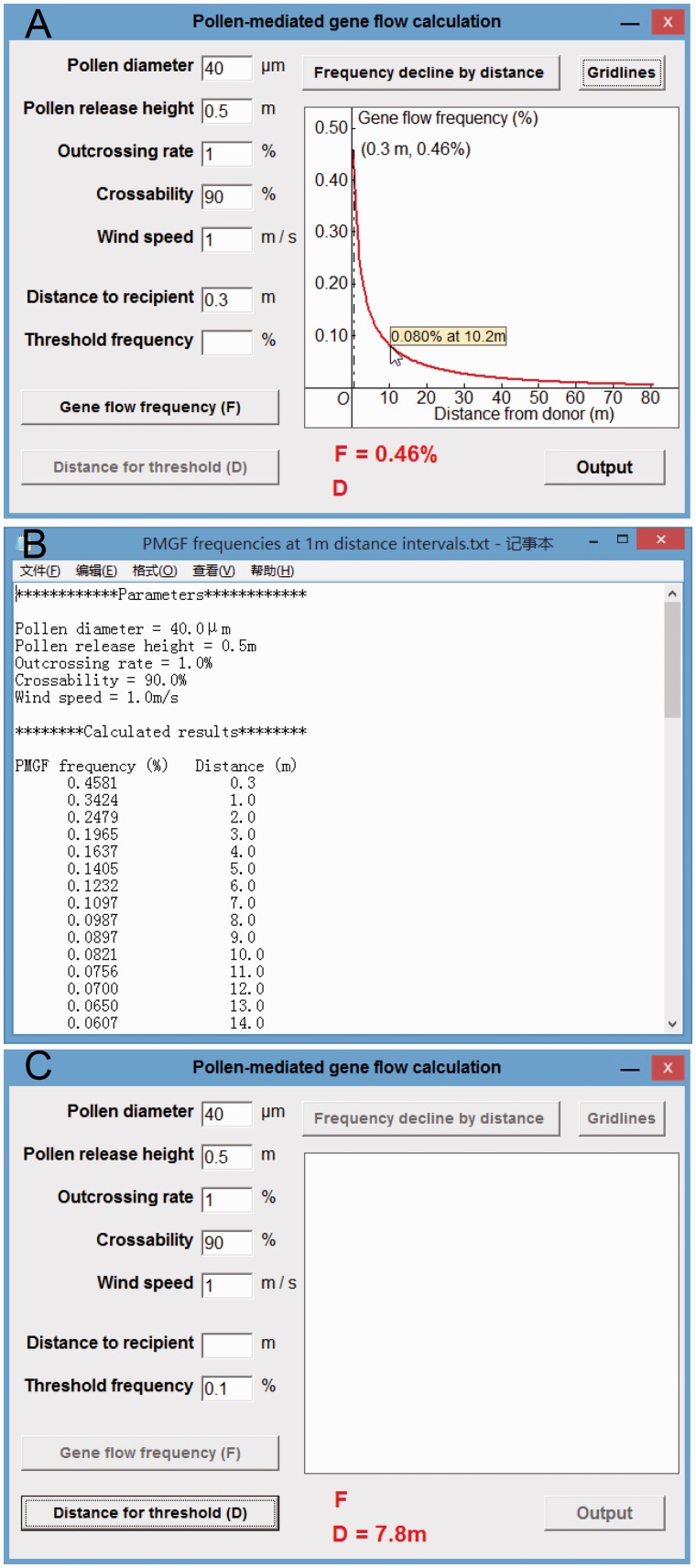
Different functions of the calculating tool for PMGF. (A) the display of a particular frequency (0.08 %) at a distance interval (10.2 m) in a floating window at the mouse cursor on the PMGF frequency curve; (B) the content of an output file showing the exported values of series PMGF frequencies (%) at every 1 m distance intervals (m); (C) the value of isolation distance (7.8 m) determined by the threshold PMGF frequency (0.1 %).

Pollen-mediated gene flow frequencies at various distances along the frequency curve (red) will be displayed in a floating window when moving the mouse cursor on the curve shown in the plotting field after clicking the ‘Frequency decline by distance’ button (Fig. 2A). An output file (PMGF frequencies at 1 m distance intervals.txt) with PMGF frequency (%) and distance (m) along the curve at the distance intervals of 1 m will be generated and exported to the folder containing the calculating tool, by clicking the ‘Output’ button (at the lower-right corner) (Fig. 2B). In addition, the spatial isolation distance for a particular threshold PMGF frequency can be calculated and displayed when clicking the ‘Distance for threshold (*D*)’ button (at the lower-left corner) after all the required parameters and a particular ‘Threshold frequency’ (variable) are included (Fig. 2C, at the lower side).

### Case studies: crop-to-crop gene flow

*PMGE frequencies of rice*. Using rice crop-to-crop gene flow as an example, we estimated PMGF frequencies of this crop by including five parameters obtained from published results: pollen diameter to be 40 μm, pollen release height to be 0.5 m, outcrossing rate to be 1 %, crossability as 90 % and wind speed to be 1 m s^−^^1^ (Bae *et al.* 2013; Wang *et al.* 2016).

**Figure 3:**
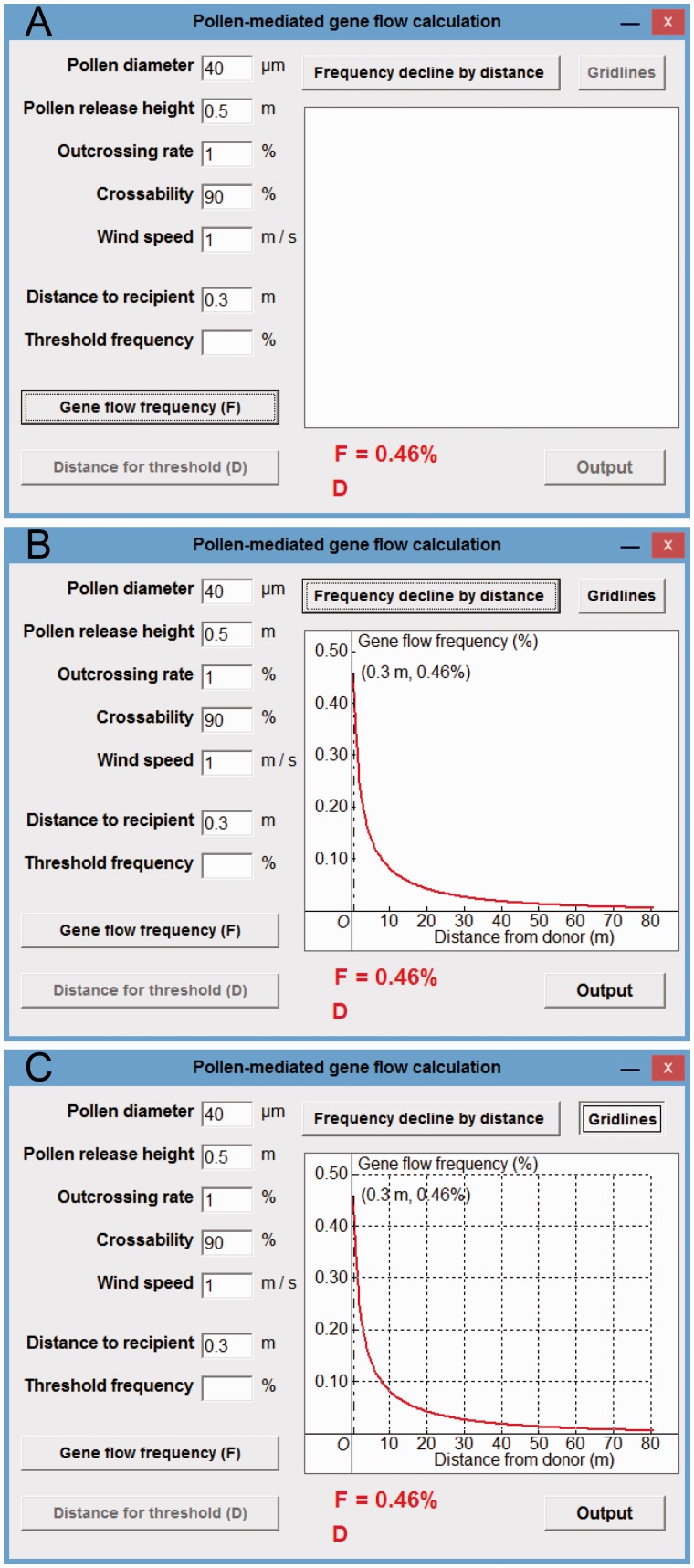
Gene flow in rice case study. (A) The display of a PMGF (PMGF) frequency (*F* = 0.46 %) at 0.3 m; (B) the display of PMGF frequencies at different spatial distances (0.3–80 m); (C) the display of gridlines.

After the inclusion of the five parameters (Fig. 3A) and distance to recipient (e.g. 0.3 m) in the respective textboxes, and clicking the ‘Gene flow frequency (*F*)’ button, a calculated PMGF frequency (*F* = 0.46 %) for the target rice recipient at a given spatial distance interval (0.3 m) was displayed in the window (Fig. 3A, at the lower side). When clicking the ‘Frequency decline by distance’ button, a series of calculated PMGF frequencies (0.46–0.0028 %, as a curve) for a given spatial-distance range (0.3–80 m) was displayed in the plotting field (Fig. 3B, at the right). When clicking the ‘Gridlines’ button, gridlines for different distance intervals (m) and PMGF frequencies (%) were provided in the plotting field (Fig. 3C). Because this tool is based on the deterministic model and include determined parameters for PMGF calculation, the calculated PMGF frequencies are determined for different distances, without confidence intervals. The model predicted PMGF frequencies (0.46, 0.32, 0.22 and 0.12 %) at the distance intervals of 0.3, 1.2, 2.4 and 6 m based on the calculating tool are similar to those (0.30–0.97, 0.05–0.33, 0.02–0.22 and 0.02–0.07 %) obtained from the PMGF experiments at the corresponding distance intervals (see Table 3 in Bae *et al.* 2013).

*PMGE frequencies of wheat*. For the wheat crop-to-crop gene flow case study, we calculated PMGF frequencies of this crop based on the following five parameters obtained from published results: pollen diameter to be 50 μm, pollen release height to be 0.5 m, outcrossing rate to be 0.5 %, crossability to be 90 % and wind speed to be 4.5 m s ^−^ ^1^ (Beckie *et al.* 2011; Wang *et al.* 2016).

**Figure 4:**
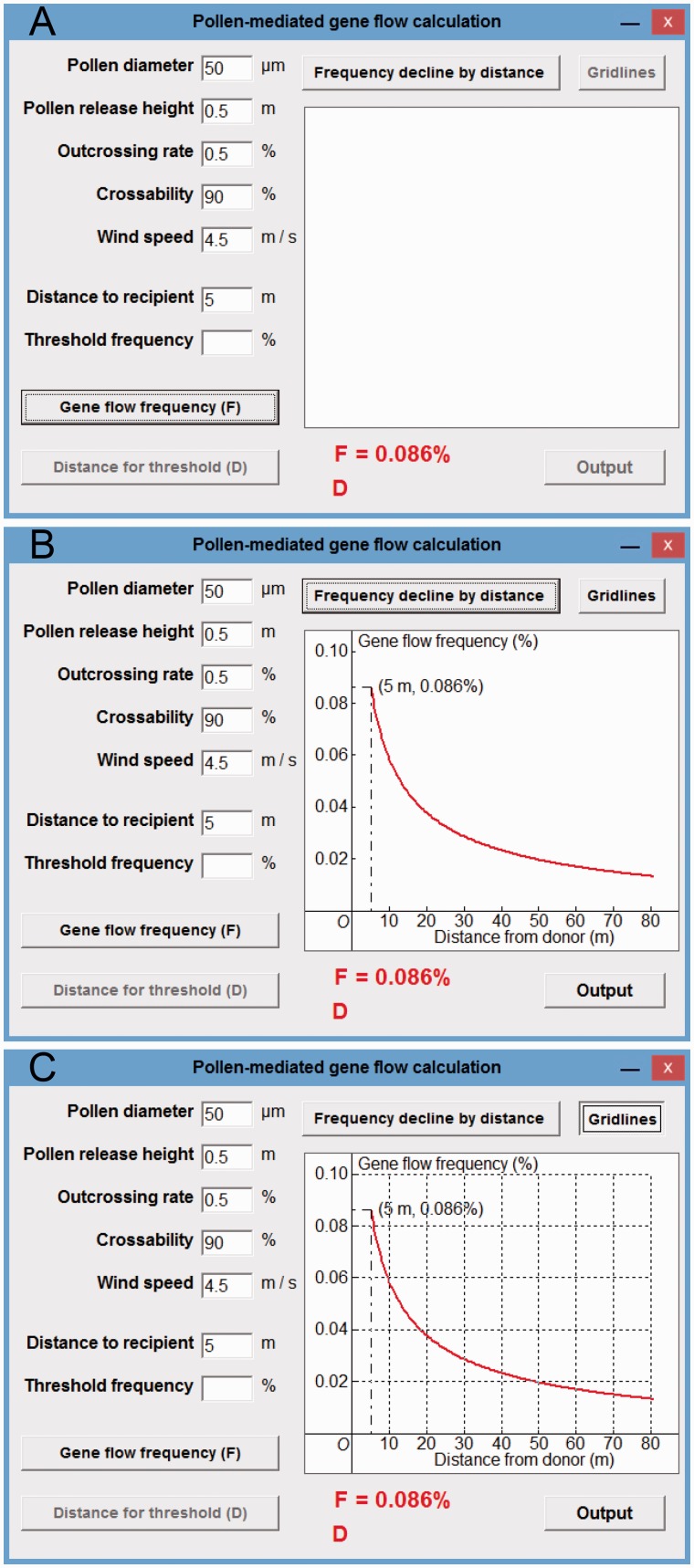
Gene flow in wheat case study. (A) The display of a PMGF frequency (*F* = 0.086 %) at 5 m; (B) the display of PMGF frequencies at different spatial distances (5–80 m); (C) the display of gridlines.

A PMGF frequency (*F* = 0.086 %) for the target wheat recipient at a given spatial distance interval (e.g. 5 m) was calculated and displayed by the tool (Fig. 4A, at the lower side) after including the five parameters (Fig. 4A) and distance to recipient (e.g. 5 m) of wheat and clicking the ‘Gene flow frequency (*F*)’ button. A series of calculated PMGF frequencies (0.086–0.013 %) for a given spatial-distance range (5–80 m) was shown as a curve in the plotting field (Fig. 4B, at the right) when clicking the ‘Frequency decline by distance’ button. Gridlines will be shown in the plot field (Fig. 4C) by clicking the ‘Gridlines’ button. The model predicted PMGF frequencies (0.086, 0.058, 0.038 and 0.029 %) at the distance intervals of 5, 10, 20 and 30 m based on the calculating tool are similar to those (0.07–0.09, 0.05–0.07, 0.03–0.04 and 0.01–0.02 %) from the field experiments at the corresponding distance intervals (see Fig. 5 in Beckie *et al.* 2011).

**Figure 5:**
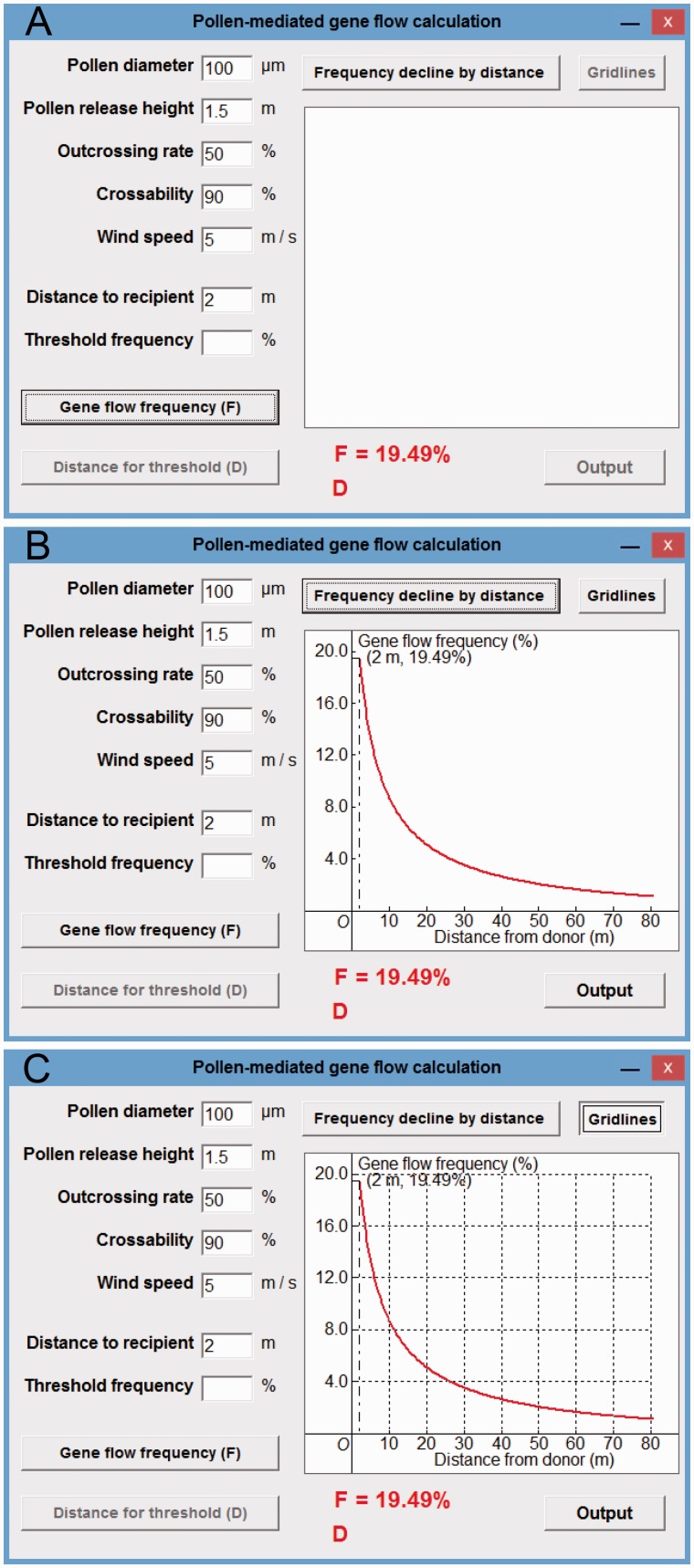
Gene flow in maize case study. (A) The display of a PMGF frequency (*F* = 19.50 %) at 2 m; (B) the display of PMGF frequencies at different spatial distances (2–80 m); (C) the display of gridlines.

*PMGE frequencies of maize*. Using maize crop-to-crop gene flow as an example, we calculated PMGF frequencies of this crop by the five parameters as follows: pollen diameter to be 100 μm, pollen release height as 1.5 m, outcrossing rate to be 50 %, crossability to be 90 % and wind speed to be 5 m s ^−^ ^1^ (Weekes *et al.* 2007; Wang *et al.* 2016).

After including the five parameters (Fig. 5A) and distance to recipient (e.g. 2 m) for maize, and clicking the ‘Gene flow frequency (*F*)’ button, a calculated PMGF frequency (*F* = 19.5 %) for the target maize recipient at a given spatial distance interval (2 m) was displayed (Fig. 5A, lower side). When clicking the ‘Frequency decline by distance’ button, a series of calculated PMGF frequencies (19.5–0.70 %, as a curve) at a range of distance intervals (2–80 m) was displayed in the plotting field (Fig. 5B, at the right). Gridlines will be displayed in the plot field (Fig. 5C), when clicking the ‘Gridlines’ button. The model predicted PMGF frequencies (19.5, 13.2, 8.7 and 3.5 %) at the distance intervals of 2, 5, 10 and 30 m based on the calculating tool are similar to those (21–22, 12–13, 7–8 and 4–5 %) obtained from the field experiments at the corresponding distance intervals (see Fig. 2 in Weekes *et al.* 2007).

Altogether, these results from the rice, wheat and maize gene flow case studies indicated that the calculating tool can generate relatively reliable PMGF frequencies for plant species.

## Discussion

We constructed a tool to calculate the frequency of PMGF based on the quasi-mechanistic PMGF model (Wang *et al.* 2016) that was selected among a large number of published PMGF models. The selection of the quasi-mechanistic model of Wang *et al.* (2016) to construct the PMGF calculating tool is due to the reason that this model can provide PMGF predictions with a relatively high level of accuracy by the inclusion of both biological and climatic parameters. Furthermore, the model-based PMGF calculating tool has an easy-operating interface due to the practical design of a human–computer dialog system, which can be easily handled by any users who do not have sufficient knowledge on mathematical modelling. To our knowledge, this is the first available calculating tool that can be used to estimate PMGF frequencies of a pollen-donor species/variety to its pollen recipients at a given spatial distance(s) based on a PMGF mathematical model. The advantage of such a calculating tool is its simplified procedure of model-based prediction for PMGF frequencies, which makes the complicated theoretical PMGF modelling applicable. This applied feature has not been achieved by the other available PMGF modelling studies that only provide mathematical equations (e.g. Klein *et al.* 2006; Wang and Yang 2010; Hu *et al.* 2014). Other types of published PMGF models may also be transformed into practical tools for calculating PMGF frequencies. Yet, it is necessary to fully understand the underlying mechanisms of the models.

The calculating tool includes four biological parameters: pollen diameter, pollen release height, outcrossing rate and crossability, in addition to a climatic parameter. These biological parameters can be measured directly from the target plant species/varieties at the field sites where PMGF frequencies need to be obtained. The required climatic parameter (wind speed) can also be measured simultaneously at the sites during the period when PMGF frequency estimation is required. That is to say, the prediction of PMGF frequencies can be relatively easily achieved at any target field sites or in the particular environment, provided that the required parameters are available through field or laboratory experiments. It is not necessary to generate parameters by conducting a specific PMGF experiment. Therefore, the calculating tool from this study can facilitate the prediction of PMGF frequencies with faster and more effective characteristics, although it is only suitable for wind-pollination plants.

To examine the predicting power of the calculating tool, we compared the crop-to-crop PMGF frequencies generated from this model-based tool and those obtained from published PMGF experiments, using rice, wheat and maize as case studies (Weekes *et al.* 2007; Beckie *et al.* 2011; Bae *et al.* 2013). We found that the predicted PMGF frequencies are similar to those from field experiments. The results indicate that the calculating tool has a relatively strong predicting power for estimating PMGF frequencies of different plant species under various environmental conditions where the required parameters are obtained. Apparently, this calculating tool is designed to estimate PMGF frequencies from a donor field to a recipient field in pure stands, which may not be suitable for donor and recipient plants that are grown in mixture, such as different types of trees in forests. In addition, it is also necessary to point out that this tool does not include parameters such as air temperature and relative humidity that may also affect PMGF as suggested by Rong *et al.* (2010). This is due to our limited understanding of the contribution of these parameters to PMGF. Future studies should be carried out to generate knowledge on the intimate relationship of parameters, such as air temperature and relative humidity, with PMGF for designing an improved PMGF calculating tool with increased accuracy.

This calculating tool has relatively wide applications associated with PMGF. The most pertinent use of this tool is to estimate transgene flow frequencies from GE crops to their non-GE counterparts and to wild/weedy relative species, which is important to assess social-economic and particularly environmental impacts caused by transgene flow as discussed previously (Ellstrand 1992, 2003; Yong and Kim 2001; Lu and Snow 2005; Loureiro *et al.* 2009; Lu and Yang 2009). If a transgene can move from a GE crop to its wild/weedy relatives at a comparatively high frequency (Song *et al.* 2003*a*; Chen *et al.* 2004; Weekes *et al.* 2005) and bring considerable fitness effects (benefit or cost) to the wild or weedy relatives (Yang *et al.* 2011; Li *et al.* 2016), rigorous biosafety measures should be taken to minimize the undesired environmental consequences (Lu and Snow 2005; Lu and Yang 2009; Lu *et al.* 2016).

In a coexistence agro-ecosystem, the determination of spatial isolation distances between GE and non-GE crops is critical to reduce transgene flow frequencies to a legally permitted threshold for non-GE crops (Devos *et al.* 2009). Currently, the spatial isolation distance is determined essentially based on PMGF experiments (Rong *et al.* 2007; Sanvido *et al.* 2008; Langhof *et al.* 2010). The PMGF experiments require a great amount of financial and labor inputs. The calculating tool established in this study can facilitate the determination of spatial isolation distances between the coexisting GE and non-GE crops based on the required threshold PMGF frequencies. For example, the isolation distances between GE and non-GE maize were determined as 50 m for an allowed threshold of LLP of transgenes (0.9 %) based on PMGF experiments (Sanvido *et al.* 2008; Langhof *et al.* 2010). Similarly, the PMGF frequency calculated using our tool is 0.86 % for maize at the isolation distance of 50 m (under a normal wind speed of 3 m s ^−^ ^1^), which is about the same as that proposed by Sanvido *et al.* (2008) and Langhof *et al.* (2010). This indicates that our PMGF calculating tool can be used in determining the isolation distance for the coexisting GE and non-GE crops.

In addition, our PMGF calculating tool can also be applied to determine the isolation distances for the production of certified seeds between field plots to guarantee the purity of the bred seeds used for agricultural production. For example, according to the criteria of different countries for certified seed production, the proposed allowance (threshold) for the mixture of undesired seeds for rice is 0.23 % and for maize is 0.50 % (see Table 2 in Zhang *et al.* 2011). On the other hand, Yong and Kim (2001) suggested the minimum spatial isolation distance to reach the allowed mixture of undesired seeds for different crops, which is 3 m for rice and 200 m for maize. To examine whether the proposed isolation distances (3 and 200 m) meet the criteria of allowed mixture (%) for rice and maize in seed production, we used this tool to calculate the frequencies of PMGF from undesired sources of crops. Consequently, the calculated frequency is 0.23 % for rice at the isolation distance of 3 m and 0.21 % for maize at the isolation distance of 200 m under a wind speed of 5 m s ^−^ ^1^, which are within the threshold of seed mixture for rice and maize seed production, respectively. This demonstrates that our PMGF calculating tool can be applied to determine the isolation distances of certified seed production for different crop species, provided that the parameters are obtained at the field sites where the certified seeds are produced. In addition, the prediction of PMGF frequencies using the PMGF calculating tool may also be useful for the studies of evolutionary and conservation biology of wild plant species. As an evolutionary driving force, gene flow plays an important role in influencing evolution of plant species, particularly for endangered wild species (Ellstrand *et al.* 2013; Ellstrand 2014). For example, the extensive gene flow mediated by pollination from cultivated rice to wild rice (*Oryza rufipogon*) that contains valuable genetic diversity for rice breeding may result in considerable losses of genetic integrity or even local extinction of this species (Song *et al.* 2003*b*). If PMGF frequencies at given spatial distances can be determined using a calculating tool, proper measures can be taken in advance to avoid such losses of genetic diversity for many wild-relative species.

## Conclusions

Based on the published quasi-mechanistic PMGF model (Wang *et al.* 2016), we constructed a tool/software that can accurately calculate PMGF frequencies of wind-pollination plant species by the inclusion of four biological and one climatic (wind speed) parameters. This tool can be easily applied by any users who are not familiar with mathematical modelling, provided that the required biological and climatic parameters are available. These parameters can be measured either directly at the target field sites/laboratories or obtained from relevant published data, without conducting a specific PMGF experiment, which makes the estimate of PMGF frequencies relatively easy and practical under different environmental conditions. Comparison between the calculated PMGF frequencies using this tool and those from published PMGF experimental data showed a good accordance between the two sets of data, suggesting the high prediction power of this tool. Therefore, this PMGF calculating tool with its easy-operating, practical and accurate features will be greatly useful for estimating transgene flow frequencies that are associated with potential social-economic and environmental biosafety impacts. This tool can also be used to determine the spatial isolation distances between GE and non-GE crops within particular threshold frequencies for GE crop presence (LLP) in the coexistence farming systems (Devos *et al.* 2009; Lu and Yang 2009). In addition, this tool can also be used to facilitate the determination of proper spatial isolation distances between field plots for producing certified crop seeds to guarantee the seed purity by maintaining the mixture of undesired seeds from PMGF within the allowed threshold (Zhang *et al.* 2011).

## Sources of Funding

This work is supported by the Natural Science Foundation of China (31330014) and the National Program of Development of Transgenic New Species of China (2016ZX08011-006).

## Contributions by the Authors

L.W. established and tested the calculating tool, and wrote the manuscript. B.-R.L. designed the study and wrote the manuscript.

## Conflict of Interest Statement

None declared.
